# Can Viewing Modality Affect Frontal Mandibular Bone Height Measurement? A Comparison Between 3D Digital Imaging and Communications in Medicine Viewer and Printed Portable Document Format Cone Beam Computer Tomography Reports

**DOI:** 10.3390/dj13010022

**Published:** 2025-01-03

**Authors:** Michael Solomonov, Yoav Shapinko, Ella Lalum, Joe Ben Itzhak, Sapir Argaman, Matan Schottig, Amit Halpern, Nirit Yavnai, Idan Stiklaru

**Affiliations:** 1 Departments of Endodontics, Israel Defense Forces (IDF), Medical Corps, Tel Hashomer Medical Center, Ramat Gan 52621, Israel; 2 “Bina” Program, Faculty of Dental Medicine, Hebrew University of Jerusalem, Jerusalem 12271, Israel; 3 Departments of Orthodontics, Israel Defense Forces (IDF), Medical Corps, Tel Hashomer Medical Center, Ramat Gan 52621, Israel; 4 Medical Corps, Israel Defense Forces (IDF), Ramat Gan 52625, Israel; 5 Department of Community Dentistry, Faculty of Dental Medicine, Hebrew University, Hadassah Ein Kerem Campus, Jerusalem 91120, Israel

**Keywords:** bone height, bone measurement, CBCT, DICOM, digital radiography

## Abstract

**Objectives:** Buccal cortical bone dimensions are crucial in dental radiology, as they impact orthodontic treatment outcomes. Changes in alveolar bone dimensions can result in malocclusion and require interdisciplinary approaches for correction. The accurate quantification of buccal bone dimensions is crucial for appropriate treatment planning and avoiding medico-legal issues. This study aimed to compare buccal bone height measurements between three-dimensional (3D) digital imaging and communications in medicine (DICOM) data and portable document format (PDF) cone beam computer topography reports for mandibular frontal teeth, testing the hypothesis of no difference in values between the two modalities. **Methods:** Each of the five observers performed a total of 720 height measurements (360 by DICOM and 360 by PDF), yielding a total of 3600 measurements overall. **Results:** Compared with the DICOM format, using PDF files was associated with a significantly greater rate of inability to carry out the measurements (8.8% vs. 3%, respectively, *p* < 0.001, chi-square). The average buccal bone height measured in the DICOM was 11.51 mm, which was significantly greater than the 10.35 mm measured in the PDF (*p* < 0.001). The mean height measured by the DICOM was consistently greater than that measured by the PDF, with highly significant differences in the findings of four of the examiners (*p* < 0.001). **Conclusions:** Viewing modality significantly affected the height of the buccal bone in the frontal mandibular area. Compared with the generated PDF reports, the 3D DICOM viewer performed better than the printed PDF and enabled more measurements in the target area.

## 1. Introduction

The entire volume of a cone beam computed tomography (CBCT) scan must be systematically assessed and reported upon [1]. It is considered good standard practice to evaluate the complete dataset acquired in a CBCT scan in the sagittal, axial, and coronal planes and poor practice to focus solely upon a limited area of interest [2]. Digital Imaging and Communications in Medicine (DICOM) has become the gold standard format for image datasets, such as CBCT-based data. A wide range of software for viewing DICOM files and for exporting sections or images in other formats is commercially available for application to diverse measurements [3]. However, many general practitioners and specialists who refer patients to CBCT continue to rely upon computer-generated reports in a single or a double plane, which is arbitrarily divided into slices in a preset interval PDF [4]. This reliance upon a generated report instead of viewing the entire scanned region in a multiplanar 3D DICOM viewer may cause loss or distortion of important data (e.g., if a slice has not been made within the longitudinal axis of a tooth, it may be unable to detect incidental findings, or when the report does not contain all of the scanned data), thus leading to incorrect or incomplete decision-making [5]. In addition, the resolution of the print is lower and depends upon other factors, such as the printer quality.

An important field of interest in dental radiology involves the dimensions of the buccal cortical bone plates both before and after orthodontic treatment [6]. Precise measurements of alveolar bone thickness and height are of significant clinical importance. For example, reductions in alveolar bone dimensions have been shown to be linked with labial tooth movements, especially flaring, tooth rotations, ectopic positions of the teeth, and anatomical variabilities of the roots, particularly in the buccolingual dimension, which may require an interdisciplinary treatment approach to correct the damage [7]. Other factors, such as pretreatment recession, incisor proclination, thin buccal cortical plates, and thin buccal soft tissue, including the wash-board effect, might require more in-depth examination. Adult or periodontally compromised patients may require particular extra caution, which is highly important for bone evaluation before and after treatment. Every change in bone level may be critical for tooth prognosis; therefore, evaluation and assessment are crucial for decision-making [8,9,10].

As teeth are moved orthodontically, the alveolar bone in the direction of the applied force undergoes constant bone turnover [11] driven by the activity of osteoclasts, which decreases the density of the bone. An increase in osteoclastic activity and a reduction in bone density are referred to as the regional acceleratory phenomenon (RAP) [12]. Although this phenomenon is temporary and considered transient, its ongoing consequences must be correctly interpreted and understood. CBCT scanners identify an object by its density. For example, buccal bone undergoing RAP would appear less clear and more lucent.

The angle at which the image plane meets the bone can also cause thin bone to appear thicker or thinner than it actually is [6]. Thus, the correct quantification and determination of buccal bone thickness and height may be misinterpreted in both directions.

In addition, the dimensions of the mandibular buccal bone height remain important for the prognostic periodontal evaluation of teeth, especially following orthodontic treatment. Underestimation of the true dimensions around the orthodontically moved teeth might lead to serious errors in decision-making and even result in irreversible unwarranted treatment procedures, such as tooth extractions. Additionally, the misinterpretation of existing bone thickness, particularly after orthodontic treatment, may lead to an inappropriate evaluation of the overall quality of the treatment, incorrect estimation of the final teeth positions, and an inaccurate assessment of the periodontal prognosis for these teeth, potentially resulting in undesired medico-legal claims. Claims based solely on PDF prints may lead to misleading conclusions and, consequently, unnecessary lawsuit issues.

The aim of this study was to investigate the possible difference in buccal bone height measurements in the mandibular frontal teeth area (canine-to-canine) between those made with DICOM-retrieved data and those made with PDF data. The null hypothesis was that there is no difference in values between these two modalities.

## 2. Materials and Methods

This study received ethical approval from the Institutional Review Board of the Israel Defense Force Medical Forces (approval number #2186-2021, date of approval: 21 March 2021) and was conducted in full compliance with all relevant ethical guidelines. Owing to the anonymized nature of the data, the requirement for informed consent was waived.

A team of five observers, comprising an orthodontist, an orthodontics postgraduate student, an endodontics postgraduate student, and two general dental practitioners, participated in this study. They randomly selected cone-beam computed tomography (CBCT) scans of the mandibular regions of 60 patients, resulting in a total of 360 teeth being examined (n = 360). These scans were retrieved from the institutional database. The scanning parameters were standardized to include a medium field of view, a voltage of 90 kV, and a voxel size of 150 micrometers, with an average radiation dose of 1137 mGy*cm^2^.

**Figure 1:**
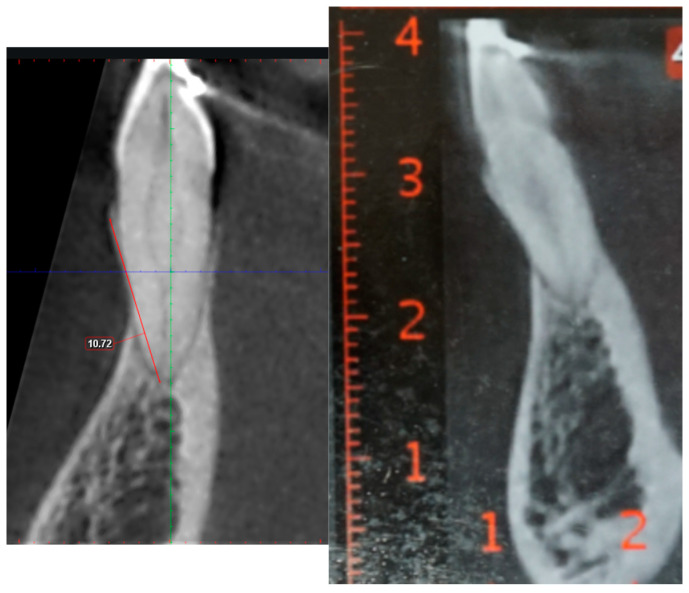
The buccal bone heights in the 3D DICOM and the PDF are similar.

The observers evaluated the selected CBCT scans twice: first by analyzing the original Digital Imaging and Communications in Medicine (DICOM) files via Planmeca Romexis Viewer software version 6.4.7 (Helsinki, Finland) and then, one month later, by assessing printed PDF reports derived from the same scans. During both evaluations, the observers measured the diagonal distance in millimeters from the root apex of a designated tooth to the most coronal and buccal aspect of the alveolar bone (as illustrated in Figure 1). For the DICOM data, the measurements were made via a built-in software ruler, whereas for the PDF reports, a standard hand ruler was used. In cases where either the root apex or the buccal bone plate tip was not clearly visible, the measurement was categorized as “missing”.

This study also included a comparison of the pooled height measurements between the two formats (PDF and DICOM) to evaluate potential discrepancies. The required sample size was calculated via WinPepi version 11.65 software, assuming a significance level of α = 0.05 and anticipating a 2 mm difference between measurement methods.

Before data collection, all observers underwent standardized training conducted by the investigator, IS, to ensure consistent measurement techniques. The training involved the evaluation and measurement of buccal bone height in 20 randomly selected frontal mandibular CBCT scans. The measurements were first performed via the built-in Romexis software version 6.4.7 ruler and subsequently repeated with a hand ruler on printed PDF reports. This calibration process aimed to minimize variability and ensure methodological consistency across observers.

To assess the reliability of the measurements, both interobserver and intraobserver agreement were evaluated. Interobserver reliability was determined using intraclass correlation coefficients (ICCs), which indicated excellent agreement for measurements derived from both PDF files (ICC = 0.949, *p* < 0.001) and DICOM files (ICC = 0.941, *p* < 0.001). The intraobserver reliability data are summarized in Table 1, detailing individual observer comparisons between the DICOM and PDF files.

### Statistical Analysis

All analyses were performed via SPSS software version 28 (IBM, North Castle, NY, USA). Categorical variables are presented as percentages, and rate comparisons were performed via the chi-square test. Continuous variables (height in mm) were normally distributed. To evaluate differences between groups, the chi-square test was employed for categorical variable comparisons, ensuring that the observed frequencies were statistically tested against expected frequencies. For continuous variables, mean comparisons were conducted using an independent t test, which allowed for the evaluation of whether the means of two independent groups differed significantly. Statistical significance was set at a threshold of α = 0.05. This robust approach ensured a comprehensive and reliable analysis of the dataset, facilitating meaningful interpretation of the results.

## 3. Results

Each observer performed 360 measurements of buccal cortical plate height, once by using the DICOM viewer and again by using the PDF files, yielding a total of 720 measurements per observer, or 3600 measurements in total. The number of times each observer was unable to measure the height (due to inability to observe the root apices and/or the tip to the buccal cortical plate) was significantly greater for the PDF-derived data than for the DICOM-derived data (8.8% of the measurements compared to 3%, respectively, *p* < 0.001, chi-square test).

The mean pooled height of the buccal bone measured with DICOM was 11.51 mm, whereas that measured with PDF was 10.35 mm, which was significantly lower (*p* < 0.001) (Table 1). Each observer separately revealed that the mean height was always greater in the DICOM measurements than in the PDF measurements and that the difference between the methodologies was significantly greater (*p* < 0.001) for four of the five observers (Table 1).

## 4. Discussion

Cone-beam computed tomography (CBCT) was first introduced into practical dentistry in the 1990s. Since then, it has become the standard method for three-dimensional radiographic imaging in dental and maxillofacial radiology, with a broad range of applications in this area [13,14]. The technology has been adapted for other fields of medicine, such as orthopedics [15], otolaryngology (ENT) [16], interventional radiology (angiography) [17], and neurosurgery [18]. The introduction of CBCT marked a significant advancement in the field of dental imaging, as it provided three-dimensional views of the craniofacial complex with much lower radiation doses than conventional CT scans [19].

In 1998, Mozzo et al. [20] introduced the first commercial CBCT unit, which was specifically designed for dental use. This technology quickly gained popularity because of its ability to produce detailed 3D images, which are essential for accurate diagnosis and treatment planning in various dental specialties, including endodontics, orthodontics, and oral surgery. Since then, CBCT has undergone several technological advancements, leading to improved image quality, reduced radiation exposure, and more compact and affordable units.

The applications of CBCT in dentistry are broad and cover various specialties:

Endodontics: In endodontics, CBCT is primarily used for the diagnosis and management of complex root canal systems, for detecting periapical pathologies, for assessing root fractures, and for evaluating the success of previous endodontic treatments. The importance of CBCT evaluation has been demonstrated in cases of external cervical resorption (ECR) [21], A new classification for ECR was launched on the basis of 3D evaluation [22]. CBCT is considered mandatory in the preparation and planning of modern apical surgery [23]. The use of guided endodontics for calcified canals requires a CBCT scan for cast fabrication and has been a growing field in recent years. CBCT has been shown to improve clinician diagnosis, decision-making and confidence in dealing with traumatic dental injuries [24].

Implantology: Historically, implantologists were the first adopters of this X-ray technology [25].

In this field, CBCT plays a key role in assessing bone density, height, and width—critical factors for successful implant placement. It also aids in identifying essential anatomical structures, such as the inferior alveolar nerve, maxillary sinus, and nasal cavity, helping to reduce the risk of surgical complications [25].

Oral and maxillofacial surgery: CBCT provides detailed images of the maxillofacial region, aiding in the diagnosis and management of fractures, cysts, tumors, and other pathologies. It is also used in the planning of reconstructive surgeries and in the assessment of temporomandibular joint disorders.

Many surgeons still rely upon printed PDF reports, which are simpler and more rapid and do not require additional knowledge and training in the use of the software. For most surgical procedures, such as implant placement or tooth extraction, gross evaluation, such as that reported on a PDF report, might suffice. However, for many other procedures and for the evaluation of prognosis, such as periodontal bone dimension estimation a more precise quantitative assessment is usually needed [26].

Periodontics: In periodontics, CBCT is used to evaluate the extent of periodontal bone loss, assess the proximity of vital structures, and plan regenerative procedures. It is particularly useful in cases where traditional radiographs are insufficient to provide a clear view of the bone architecture [27].

Orthodontics: CBCT is invaluable in orthodontics for assessing craniofacial morphology, analyzing airway space, and planning orthognathic surgery. It enables measurements of dental and skeletal structures, aiding and monitoring in the development of customized treatment plans. CBCT is especially warranted when it offers advantages to the patient or alters the treatment outcome in comparison with traditional imaging methods. Among these imaging modalities, the most recognized indications in orthodontics are for the evaluation of craniofacial abnormalities, sinus anatomy or pathology, pharyngeal airway, and root resorption, as well as for the assessment of impacted and ectopic teeth, mini-implant sites, and the cortical bone plate, and for planning and evaluating orthognathic surgery [28,29].

CBCT usage is a valuable tool in modern orthodontics, providing 3D information that 2D methods cannot satisfy. CBCT imaging allows for the segmentation of each tooth, including the roots, which allows for the precise planning of tooth movements, including root torque [30].

Moreover, a digital workflow enhances collaboration and enables the sharing of virtual records among dental specialists in a multidisciplinary setting, promoting clear and open communication with patients. When a patient needs extensive treatment, creating an accurate and thorough diagnosis is essential.

During orthodontic tooth movement, the alveolar bone undergoes constant bone turnover in the direction of the applied force [9]. Although there are multiple treatment modalities, the direction of mandibular incisor movement is toward the buccal bone plate in most orthodontic cases, especially in cases of lower frontal crowding release without tooth extraction, during the relief of a deep occlusal curve (Spee curve), during long-term usage of intermaxillary elastics (particularly Class II elastics in cases of corrections of dental Class II malocclusions, which are mainly based on mandibular mesialization), or all of the above. In such cases, there is increased potential for anterior inclination of the lower incisors, which may not necessarily involve bodily movements, sometimes causing unwanted pressure on the crestal bone surface and subsequent horizontal bone resorption. Proper orthodontic mechanics should be planned to create appropriate tooth movements, ensuring physiological bone turnover while preventing or minimizing these undesired side effects.

In conclusion, a balanced relationship between soft and hard tissues can be attained, resulting in facial harmony. This is a crucial element in maintaining ongoing informed consent, which helps lower the risk of legal claims.

Medicolegal issues:

Medicolegal issues in dentistry, particularly concerning bone levels, revolve around a few critical topics. These issues are important for dental practitioners to understand to minimize legal risks and provide the highest standard of care to their patients. Among the most popular issues are accurate diagnosis, bone quantity and quality assessment, patient communication and information, documentation, and patient follow-up. To form an opinion on whether the dentist’s conduct fell within the acceptable standard of care, experts review the patient’s dental records, imaging, treatment plans, and other relevant documentation [31].

In several cases, the clinician is required to provide proof of the final posttreatment bone level around the previously moved teeth, especially from the buccal side in the lower incisor area, which is not impaired during orthodontic treatment, or that alterations in the bone level have no clinical significance and do not impact the short- or long-term prognosis. Therefore, qualitive evaluation of the buccal bone level might not be sufficient; hence, precise quantitative estimation of periodontal bone support is crucial. In several cases, small differences between the estimated bone levels may mislead the clinician into unwanted conclusions regarding the future periodontal prognosis of the moved teeth, resulting in unnecessary periodontal procedures or even tooth extractions. For example, an incorrect estimation of only 1–2 mm of bone might dramatically change the prognosis of a tooth and the desired orthodontic treatment plan [32,33]. The anterior mandible has been extensively studied in terms of its anatomy, structure, and embryology because of its diverse variations and distinct anatomical, neurological, and radiological characteristics [34,35].

For this study, we specifically selected mandibular frontal teeth (canine-to-canine) for analysis because of their clinical relevance in treatment planning and their susceptibility to periodontal and orthodontic concerns. The results reveal a significant difference in the mean buccal bone height values obtained from the two measurement modalities. The measurements derived from the 3D DICOM format were significantly greater than those obtained from the printed PDF reports, with mean values of 11.51 mm and 10.35 mm, respectively (*p* < 0.001). This difference of 1.16 mm, while potentially negligible in patients with a healthy periodontium, could have critical clinical implications in patients with existing periodontal loss. For such patients, a reduction in buccal bone height measurements might alter the prognosis of teeth from “fair” to “hopeless,” directly impacting treatment decisions.

**Figure 2:**
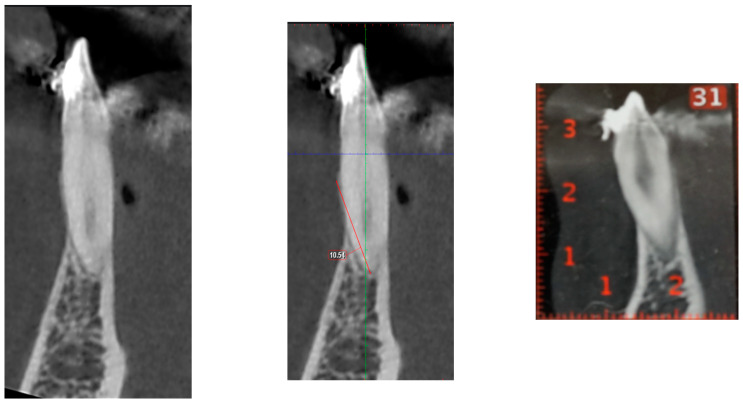
Compared with that in the PDF, the height of the buccal bone in the 3D DICOM is much greater.

The observed variability in the data was also notable, with standard deviation values ranging between −2.86 mm and 3.51 mm. This range indicates that in specific samples, the actual difference in measurements could be as high as 7 mm. This discrepancy is clinically significant even in patients with healthy periodontal support, as it could result in misjudgment of the alveolar bone condition. To illustrate this point, Figure 1 highlights a case where the measurements from the DICOM and PDF files were relatively similar, showing minimal differences. In contrast, Figure 2 shows a case where the discrepancy between the two modalities reached a mean difference of 9 mm. Differences of this magnitude could drastically alter the treatment plan, potentially leading to over- or underestimation of periodontal health and bone support.

An additional finding of importance was the significant difference (*p* < 0.001) in the number of cases deemed nonmeasurable between the two formats. The PDF modality had 154 cases (8.8%) where height measurements could not be recorded, whereas only 54 cases (3%) with the DICOM format could be recorded. This discrepancy may have profound clinical implications. For example, nonmeasurable cases in the PDF modality could hinder accurate diagnosis or planning, particularly in orthodontic procedures that risk compromising bone support or in periodontal treatments requiring precise assessment of alveolar bone levels. The increased number of nonmeasurable cases with the PDF modality underscores the importance of using high-quality 3D imaging interpretation software to ensure accurate and comprehensive evaluations.

Since DICOM is the raw output of the CBCT scan, reviewing it involves no additional complexity or cost. However, a potential technical challenge is that the file size is relatively large and requires suitable viewing software for proper operation.

The prescription of CBCT requires judicious and sound clinical judgment. While CBCT is a valuable tool, it should not replace the need for a thorough clinical examination and traditional X-ray scans. Recently, there has been a concerning trend (published by dental practitioners where clinicians are making diagnoses and treatment decisions on the basis solely of CBCT scans). This approach overlooks the importance of a comprehensive clinical evaluation, which is essential to avoid mistakes in actual patient care. The authors have encountered numerous requests for treatment advice that includes only CBCT scans, with no accompanying clinical details. CBCT should complement—not replace—proper clinical evaluation.

The primary limitation of this study lies in the absence of a universally accepted “gold standard” for comparison, as the actual anatomical height of the measured cases was not validated through histological analysis. Without such validation, the measurements obtained from the DICOM and PDF modalities cannot be directly compared to a definitive reference, which may introduce some degree of uncertainty into the results. Future studies incorporating histological evaluations or other precise anatomical reference methods could help address this limitation and increase the reliability of imaging-derived measurements.

Another limitation is the scope of the sample, which, while adequate for the purposes of this study, may not fully represent the wide range of anatomical variations present in different populations. Specifically, age-related changes in anatomical features, such as variations in spongy bone density and cortical bone thickness commonly observed in elderly patients, were not explicitly accounted for in this research. These factors could influence buccal bone measurements and may have implications for clinical assessments in older patients. Investigating such variations presents an important opportunity for future research to expand the generalizability of findings.

Furthermore, this study did not explore potential ethnic differences in anterior mandibular bone characteristics, which may affect CBCT interpretations and clinical decision-making. Variations in bone density, structure, and morphology across different ethnic groups could play a significant role in the accuracy and applicability of imaging measurements. Addressing this gap would require a more diverse sample population and could be effectively pursued through a multicenter study involving diverse ethnic groups. Such research would not only deepen the understanding of CBCT-based assessments but also contribute to more personalized and equitable clinical approaches.

## 5. Conclusions

The choice of viewing modality for alveolar bone height had a significant influence on measurements in the frontal mandibular area. The 3D DICOM viewer outperformed the printed PDF files in terms of the number of measurements that could be executed. The 3D DICOM viewer also yielded significantly greater bone height measurements than those generated by the PDF reports. The superiority of 3D DICOM may be especially relevant to the avoidance of unnecessary irreversible procedures that have long-term consequences.

## Figures and Tables

**Table 1:** Differences in the measurements of buccal bone height by the 3D DICOM viewer vs. the printed PDF system.

Observer	Height (mm) in DICOM Mean ± SD (N)	Height (mm) in PDF Mean ± SD (N)	*p* Value *
Pooled five observers	11.51 ± 3.15 (1642)	10.35 ± 3.56 (1746)	<0.001
GP	10.14 ± 3.51 (333)	11.07 ± 3.35 (351)	<0.001
GP	9.57 ± 3.60 (322)	11.54 ± 3.29 (346)	<0.001
Endo postgrad	9.77 ± 3.79 (320)	11.70 ± 2.86 (347)	<0.001
Ortho	11.49 ± 3.20 (329)	11.87 ± 2.96 (338)	0.105
Ortho postgrad	10.76 ± 3.34 (338)	11.37 ± 3.20 (354)	0.014

* Independent *t* test. GP, general practitioner; Endo, endodontics postgraduate student; Ortho, orthodontist; Ortho postgrad, orthodontal postgraduate student.

## Data Availability

The data that support the findings of this study are available upon request from the corresponding author.
